# Residential Segregation and Overweight/Obesity Among African-American Adults: A Critical Review

**DOI:** 10.3389/fpubh.2015.00169

**Published:** 2015-07-02

**Authors:** Irma Corral, Hope Landrine, Marla B. Hall, Jukelia J. Bess, Kevin R. Mills, Jimmy T. Efird

**Affiliations:** ^1^Department of Psychiatry and Behavioral Medicine, Brody School of Medicine, East Carolina University, Greenville, NC, USA; ^2^Center for Health Disparities, Brody School of Medicine, East Carolina University, Greenville, NC, USA; ^3^Department of Public Health, Brody School of Medicine, East Carolina University, Greenville, NC, USA

**Keywords:** residential segregation, Blacks, African-Americans, obesity, overweight

## Abstract

The relationship between residential segregation and overweight/obesity among African-American adults remains unclear. Elucidating that relationship is relevant to efforts to prevent and to reduce racial disparities in obesity. This article provides a critical review of the 11 empirical studies of segregation and overweight/obesity among African-American adults. Results revealed that most studies did not use a valid measure of segregation, many did not use a valid measure of overweight/obesity, and many did not control for neighborhood poverty. Only four (36% of the) studies used valid measures of both segregation and overweight/obesity and also controlled for area-poverty. Those four studies suggest that segregation contributes to overweight and obesity among African-American adults, but that conclusion cannot be drawn with certainty in light of the considerable methodologic problems in this area of research. Suggestions for improving research on this topic are provided.

The prevalence of overweight and obesity is high among all Americans, and among African-Americans in particular (1–3). For example, rates of overweight/obesity among African-American women are 44% higher than among White women (1–5). Racial disparities in socioeconomic status (SES) (3, 5) and in health behaviors (e.g., diet, physical activity) contribute to racial disparities in body weight (5, 6), and African-American cultural variables such as religiosity (7, 8) and acculturation level (9) contribute as well, but these individual-level factors do not fully explain the disparities (5).

Recent research has highlighted the neighborhood context as an additional determinant of obesity and obesity disparities (5, 10–15). Such studies have identified *obesogenic environments* as contributors, i.e., neighborhoods that facilitate overweight/obesity via their paucity of parks and recreational facilities, prevalence of fast food outlets, dearth of healthy food options, and dangerousness that discourages outdoor activities (10–15). Low-SES neighborhoods have been demonstrated to be obesogenic environments that contribute to overweight and to obesity among their residents irrespective of resident race-ethnicity (11–15). Racially segregated African-American neighborhoods also meet the criteria for obesogenic environments (16) in terms of their food choices (17–20) and recreational resources (21–23). Thus, residing in a segregated, African-American neighborhood might contribute to overweight and obesity among African-Americans, and might in part explain the racial disparities in body weight that transcend low-SES (10, 16). The relationship between residential segregation and overweight/obesity, however, remains unclear (10, 16). This paper reviews the empirical studies on residential racial segregation and body weight among African-American adults in an effort to elucidate the segregation–obesity relationship.

First, we define racial residential segregation in the U.S. and highlight the various ways in which it is measured. Then, we review and compare studies of segregation and weight among African-American adults to ascertain the nature and strength of that relationship, and highlight its implications for preventing and reducing racial disparities in obesity.

## Residential Segregation

Residential segregation refers to the geographic separation of Whites from minorities (African-Americans in this case) in residential areas (24, 25). It can be measured at any geographic-level (e.g., census tracts, zip codes, counties, Metropolitan Statistical Areas/MSAs) and usually is measured at the level of census tracts or MSAs in health research (25–29). Irrespective of area-level used, segregation can be measured in several ways, including Isolation, Dissimilarity, Concentration, Clustering, Centralization, and Hypersegregation (26, 28, 29). These terms are defined in Table 1. Each of these standard, valid measures of segregation is computed by the U.S. Census Bureau, and ranges from 0 to 1, with ≥0.60 regarded as high segregation (25–29).

**Table 1:** **Dimensions and measures of residential segregation^a^ (29)**.

Dimension	Definition and measure
Evenness	The distribution of Whites vs. a minority group across residential areas resulting in mostly White vs. mostly minority neighborhoods. Interpreted as the percentage of the minority group who would have to move to achieve residential integration. Measured by the *Segregation (Dissimilarity) Index*
Isolation/exposure	The average probability that minority group members will encounter only similar others (no Whites) in their residential neighborhood. Measured by the *Isolation Index*
Concentration	The population density of segregated minority areas; the amount of physical space occupied by the segregated minority group. Measured by the *Delta Index*
Clustering	The degree to which minority neighborhoods are adjacent to each other vs. dispersed; high clustering refers to several adjacent minority neighborhoods. Measured by the *Spatial Proximity Index*
Centralization	The degree to which minority neighborhoods are located near a metropolitan area’s urban center (vs. its suburbs). Measured by the *Absolute Centralization Index*
Hypersegregation	The simultaneous occurrence of all of the above

*^a^See www.census.gov*.

The percentage of African-Americans in an area often is used as a measure of segregation as well (25–29). This crude measure lacks validity and is unrelated to the valid measures of segregation (26, 28, 29). As shown by the 10 census tracts in Table 2 for example, the percentage of African-Americans in an area can range from 32 to 55% yet the census tract might not be highly segregated, i.e., the Isolation Index can be lower than 0.60. This is because, unlike the percentage of African-Americans, valid measures of segregation take into account relative population sizes and their distribution across an area. We highlight this because (as will be shown) many studies used percent African-Americans as their measure of segregation.

**Table 2:** **Percent Blacks and Isolation Index for 10 census tracts (CT) in Pitt County, NC, USA**.

CT	*N*	Land area (square miles)	People per square mile	Blacks *N* (%)	Whites *N* (%)	Black Isolation Index
601	6,686	4.83	1,372	3,688 (55.2)	2,564 (38.4)	0.56
603	9,570	5.55	1,723	3,694 (38.6)	5,068 (53.1)	0.42
900	8,052	81.28	98	2,752 (34.2)	4,557 (56.6)	0.41
1302	5,177	3.78	1,369	1,728 (33.4)	3,154 (60.9)	0.36
1402	2,591	25.46	102	920 (35.5)	1,552 (59.9)	0.37
1500	3,315	18.65	177	1,159 (35.0)	1,950 (58.8)	0.37
1301	3,883	6.47	599	1,307 (33.7)	2,382 (61.34)	0.45
1401	4,801	12.95	371	1,982 (41.3)	2,522 (52.5)	0.50
1600	7,843	51.72	152	2,516 (32.1)	4,835 (61.7)	0.39
1900	2,889	61.87	47	1,022 (35.4)	1,768 (61.2)	0.39

*Source: http://www.usa.com/pitt-county-nc.htm*.

## Method

PubMed and PsychINFO were searched using these search terms: African-Americans, Blacks, residential segregation, racial segregation, racial composition (first set of terms), and adult obesity, adult body mass index (BMI), and adult body weight (second terms). This initial search returned 58 publications. Their abstracts then were examined by all authors; publications that were theoretical, dissertations, commentaries, did not measure segregation, focused on other racial-ethnic groups and did not contain African-Americans, or focused on children and did not include adults were excluded. Only articles that were empirical studies of adult body weight, measured segregation, and included U.S. African-American adults were retained. The final set of articles consisted of 11 publications that met all inclusion criteria.

The 11 full-length articles (10, 16, 30–38) were examined independently by the authors, each of whom completed a table detailing these article-variables: author and publication year; database (national vs. local); in-person vs. telephone interview methodology; sample size, gender, and age; definition and measurement of overweight and obesity; measure of segregation and definition of high segregation; control for neighborhood low-SES, and whether the best measure of that (i.e., percent below the federal poverty line [see Ref. (29)]) was used; and their results. Inconsistencies among the tables were discussed and articles re-analyzed until 100% agreement among the authors was reached. The 11 articles are shown in Table 3 with details on the above article-variables.

**Table 3:** **Eleven empirical studies of segregation and overweight/obesity among African-American adults**.

Reference	Database	Sample	Obesity measure and definition	Segregation measure and categories	Area-poverty controlled?	Results
Boardman et al. (10)	1990–1994 NHIS (National) in-person interview	30,891 Black adults 41.3% men All ages	Obese = BMI ≥ 30 Measured and self-reported height/weight	% Blacks in an area: ≥25% = high concentration <25% = low concentration	Yes. As % below the poverty line	Obesity prevalence higher among those in high Black concentrated areas

Chang (30)	2000 BRFSS (National) telephone interview	35,410 Whites 8,800 Blacks All ages	Overweight = BMI ≥ 25 Obese = BMI ≥ 30 Self-reported height/weight	Black Isolation Index and % Blacks in an area. Continuous, no categories	Yes. As % below the poverty line	No relationship between obesity and segregation or obesity and % Black for Blacks. Overweight increased with segregation and with %Black among Blacks. No relationship between segregation or % Black and overweight or obesity among Whites

Chang et al. (31)	2002 and 2004 SE PA Household Health Survey (Local)	6,698 adults 38.3% Blacks 7.8% Latinos All ages	Overweight = BMI ≥ 25 Obese = BMI ≥ 30 Self-reported height/weight	Black Isolation Index (Iso) and % Blacks in an area High = >60% Blacks and Iso > 0.6 Moderate = 20–59% Blacks and Iso: 0.2–0.6 Low = <20% Blacks and Iso < 0.2	Yes. As % below the poverty line	No relationship between segregation and obesity for men. For women, obesity prevalence increased with segregation and % Black

Corral et al. (16)	2000 BRFSS (National) telephone interview	11,142 Black adults 3,791 men 7,351 women All ages	Overweight and obesity together as BMI ≥ 25 Self-reported height/weight	Black Isolation Index: Low < 0.50 Moderate: 0.51–0.59 High ≥0.60	Yes. As % below the poverty line	Overweight/Obesity prevalence higher among high than low segregated, no effect for moderate segregation. No effect for gender

Do et al. (32)	1988–1994 NHANES (National) in-person interview	5,493 Whites 4,042 Blacks 3,973 Latinos 644 others Ages ≥20	BMI without categories Height/weight measured	% Blacks in an area, no categories	No. Area affluence and disadvantage were used, but area-poverty not controlled	BMI increased with % Blacks for Black men but not for Black women

Kershaw et al. (33)	1999–2006 NHANES (National) in-person interview	2,660 Black adults 1,296 men 1,364 women All ages	Obese = BMI ≥ 30 Height/weight measured	Black Isolation Index: Low ≤ 0.30 Moderate: 0.31–0.60 High > 0.60	Yes. As % below the poverty line	No relationship between segregation and obesity for men. Obesity prevalence higher among medium and high than among low segregated women

Li et al. (34)	SE PA Household Health Survey (Local) phone Interview	12,730 Whites 4,290 Blacks All ages	Obese = BMI ≥ 30 Self-reported height/weight	% Blacks in an area: ≥25% = high and <25% = low concentration	Yes. As % below the poverty line	No relationship between Black concentration and obesity for Blacks or Whites

Lim et al. (38)	NYC Community Health Survey 2002 (Local), telephone	*N* = 23,006 Whites: 39.9% Blacks: 23.1% Latinos: 23.1%	Obese = BMI ≥ 3 Self-reported height/weight	Zip-code level % Black % Latino	Yes. As % below poverty line	Increased Black residents associated with increased obesity prevalence among Blacks

Mobley et al. (35)	2001–2002 WISE-WOMAN Study (Local) in-person interview	2,692 women 60% White 13% Black 18% Latino	BMI without categories Height/weight measured	Black Isolation Index Continuous, with no segregation categories	No. Area median income and median home values used, but area-poverty was not controlled	No relationship between segregation and BMI for the women of any racial-ethnic group

Robert and Reither (36)	1986 Americans Changing Lives Survey (National)	3,617 adults 778 Black women, 396 Black men Ages ≥25	Self-reported height/weight BMI without categories	% Blacks in an area, no categories	No. Area disadvantage and Gini coefficient used, but area-poverty was not controlled	No relationship between % Blacks and BMI

Ruel et al. (37)	1986, 1989, 1994, and 2002 Americans Changing Lives Survey (National)	5,145 adults 1,727 Blacks Women only Ages 24–70 only	Self-reported height/weight BMI without categories	% Blacks in an area, no categories	No. Area disadvantage and Gini coefficient used, but area-poverty was not controlled	Increasing % Black associated with small decreases in BMI among Black women

### Data-analytic strategy

Articles were compared on their (1) definition and measurement of overweight and obesity; (2) measure of segregation and definition of high segregation; (3) measurement and control for neighborhood low-SES; (4) report of gender-specific analyses; (5) use of a local vs. national database and sample; and (6) their findings.

## Results

### Overall findings

Eight of the 11 studies (73%) reported a positive relationship between segregation and BMI/obesity/overweight; increases in segregation were associated with increases in BMI or in overweight or obesity prevalence in those studies.

### Measure/definition of overweight and obesity

Although the valid, biomedical definitions are overweight = BMI ≥ 25 and obesity = BMI ≥ 30, four studies (36%) did not use these BMI categories in data analyses. Instead, they used continuous BMI scores. Of these four, two (50%) found no relationship between continuous BMI and segregation, and two found a positive relationship for men or for women only. In the remaining seven studies, six defined obesity as a BMI ≥ 30 and one used BMI ≥ 25 to define the combined variable overweight/obesity. Six of these seven studies (86%) found a positive relationship between segregation and BMI-categorical overweight and/or obesity. Eight of the 11 studies used self-reported height and weight, and six of those eight (75%) found a positive relationship between segregation and overweight or obesity. Three studies measured height and weight, and two of those (67%) found a similar positive relationship.

### Measure of segregation

Only five of the 11 studies (45%) used a valid measure of residential segregation (e.g., the Isolation Index, see Table 1); a positive relationship was found in four (80%) of those studies. Six (55%) of 11 studies used the percentage of African-Americans in an area as their measure of segregation, with a positive relationship found in four of those studies (67%). Most studies (8 of 11 or 73%) did not define high segregation using standard, valid categories shown in Table 1. Two of the five that used Isolation Index scores used continuous scores. Only three studies that used the Isolation Index defined high segregation as scores ≥0.60; two of these found a positive relationship between segregation and overweight/obesity for women only. The six studies that used percent African-Americans as their measure of segregation either did not define the percentage that constitutes high segregation (four of the six studies), or defined high segregation as ≥25% African-Americans in a census tract or zip code.

### Control for neighborhood poverty

Seven of the 11 studies (64%) used percent of area residents below the federal poverty line as their measure of low-SES neighborhoods, and then controlled for area-poverty in their analyses. Six of those studies (86%) found a positive relationship between segregation and overweight/obesity. Of the four studies that used measures of neighborhood SES (e.g., income inequality, median home values) that are less sensitive to area-SES health disparities, two found a positive relationship and two found no effect.

### Gender analyses

Two studies had women-only samples. Both used invalid measures of segregation and used continuous BMI. Only one found increases in BMI with increases in segregation. Four studies reported gender-specific analyses; all four used valid measures, and all found a positive relationship between segregation and overweight/obesity. Two found this for women only, one found this for men only, and one found no effect for gender.

### Database/sample

Seven studies (64%) used national databases, such as the National Health Interview Survey (NHIS), National Health and Nutrition Examination Survey (NHANES), or Behavioral Risk Factor Surveillance System (BRFSS), and used large, random, national samples; six of these (86%) found a positive relationship. Of the four studies that used local (smaller) databases, half found a positive relationship and half found no effect.

## Discussion and Conclusion

Of 11 studies of segregation and overweight/obesity, 73% reported that increased segregation or high segregation was associated with increased obesity/overweight prevalence or increased BMI among African-Americans adults. The conclusion that segregation is associated with overweight/obesity cannot be drawn from that finding, however, because of the considerable problems in the measures and data-analytic strategies of the studies. Most (55% of) studies did not use a valid measure of segregation. Instead, they defined segregation as the percentage of African-Americans in an area (e.g., ≥25% of area residents), a measure known to lack validity. Others used the Isolation Index (a valid measure) but treated it as a continuous variable, without defining Isolation ≥ 0.6 as high segregation. Overall, 73% of the studies did not define high segregation using valid research categories. Moreover, only 64% used BMI categories to define obesity and overweight, only 64% included adequate controls for neighborhood-poverty in their analyses, and only 36% reported gender-specific analyses despite well-known gender differences in obesity (1–3).

Nonetheless, the 11 studies provide insights into the relationship between segregation and overweight/obesity, and highlight ways to improve this area of research. Specifically, 86% of the studies that used BMI categories to define overweight/obesity (i.e., valid measures) found a positive relationship to segregation, compared to only 50% of those that did not use BMI categories. Likewise, 80% of the studies that used valid measures of segregation found a positive relationship compared to 66% of those that used invalid measures. Similarly, 86% of the studies that used adequate controls for neighborhood poverty found a positive segregation–body weight relationship vs. 50% of those without such controls. Moreover, 86% of studies that used national databases found a positive segregation–body weight relationship compared to only 50% of those using local datasets.

Such findings suggest that inconsistencies in study results by and large reflect inconsistencies in their measures and their control of neighborhood-SES; studies tended to find a positive relationship if they used valid measures of segregation and of overweight/obesity, and also controlled for area poverty. Indeed, 100% of the studies that used valid measures of overweight/obesity and of segregation and also controlled for area-poverty found a positive segregation–obesity/overweight relationship [i.e., Ref. (16, 30, 31, 33)], but there were only four such studies. Those four lead to the tentative conclusion that residential segregation indeed may be associated with overweight/obesity among African-American adults (women in particular), but more studies with similar robust measures and controls are needed to draw that conclusion. A more firm conclusion about the segregation–overweight/obesity relationship cannot be drawn because only 45% of studies used a valid measure of segregation. However, if segregation is associated with overweight/obesity among African-American adults, then efforts to prevent and to reduce racial disparities in overweight/obesity can be improved by targeting (not African-American people but) African-American places – i.e., segregated African-American neighborhoods and their obesogenic features. These tentative conclusions must be considered in the context of the limitations of this study.

One important limitation is that we included only studies in which residential segregation was measured, irrespective of the validity of that measure. Hence, relevant studies that did not measure segregation were excluded. Two such studies (39, 40) examined racial disparities in obesity (defined as BMI ≥ 30) in racially integrated census tracts and found no African-American–White differences in obesity prevalence in integrated neighborhoods among women (39) or men (40). These two studies indirectly support the tentative conclusion that segregated African-American neighborhoods contribute to racial disparities in obesity. A second limitation is the methods sections of the 11 studies reviewed here. Details of the methods and measures (and even the sample sizes) were not provided in some articles, and hence our summary (Table 3) of their methods and measures (of neighborhood-SES in particular) might be somewhat less than accurate. In addition, we excluded studies of children from this review because the definition of overweight/obesity in children (percentile rank relative to age- and sex-matched peers) differs from the definition for adults, and a single definition was preferred. Studies of segregation and overweight/obesity among children may or may not be similar to those of adults in methodological shortcomings and findings. Despite these limitations, this review is the first of its kind, and highlights serious flaws in this area of research – use of invalid measures of segregation and of obesity foremost among those. Hence, we encourage future studies to use valid measures of segregation and of overweight/obesity to clarify the relationship between them and potentially enhance prevention efforts.

## Conflict of Interest Statement

The authors declare that the research was conducted in the absence of any commercial or financial relationships that could be construed as a potential conflict of interest.
